# Tumours of the Cerebellum

**Published:** 1908-09

**Authors:** 


**Tumours of the Cerebellum.** By JOHN WYLLIE, M.D. Pp. 109. London: H. K. Lewis. 1908.

This little book begins with a very brief description of the structure and functions of the cerebellum, and then follows an account of the symptoms, character, diagnosis, prognosis and treatment of cerebellar tumours, based on an analysis of eighty recorded cases. It is intended for senior students, and may be said to contain a fairly complete statement of the known facts about growths in this situation.

